# Novel Detection of *Coxiella* spp., *Theileria luwenshuni*, and *T*. *ovis* Endosymbionts in Deer Keds (*Lipoptena fortisetosa*)

**DOI:** 10.1371/journal.pone.0156727

**Published:** 2016-05-31

**Authors:** Seung-Hun Lee, Kyoo-Tae Kim, Oh-Deog Kwon, Younsung Ock, Taeil Kim, Donghag Choi, Dongmi Kwak

**Affiliations:** 1 College of Veterinary Medicine, Kyungpook National University, Daegu, South Korea; 2 Animal Health Center of Zoo Land, Daejeon O-World Theme Park, Daejeon, South Korea; 3 Dongin Veterinary Clinic, Daegu, South Korea; 4 Cardiovascular Research Institute, Kyungpook National University, Daegu, South Korea; Texas A&M Health Science Center, UNITED STATES

## Abstract

We describe for the first time the detection of *Coxiella*-like bacteria (CLB), *Theileria luwenshuni*, and *T*. *ovis* endosymbionts in blood-sucking deer keds. Eight deer keds attached to a Korean water deer were identified as *Lipoptena fortisetosa* (Diptera: Hippoboscidae) by morphological and genetic analyses. Among the endosymbionts assessed, CLB, *Theileria luwenshuni*, and *T*. *ovis* were identified in *L*. *fortisetosa* by PCR and nucleotide sequencing. Based on phylogeny, CLB 16S rRNA sequences were classified into clade B, sharing 99.4% identity with CLB from *Haemaphysalis longicornis* in South Korea. Although the virulence of CLB to vertebrates is still controversial, several studies have reported clinical symptoms in birds due to CLB infections. The 18S rRNA sequences of *T*. *luwenshuni* and *T*. *ovis* in this study were 98.8–100% identical to those in GenBank, and all of the obtained sequences of *T*. *ovis* and *T*. *luwenshuni* in this study were 100% identical to each other, respectively. Although further studies are required to positively confirm *L*. *fortisetosa* as a biological vector of these pathogens, strong genetic relationships among sequences from this and previous studies suggest potential transmission among mammalian hosts by ticks and keds.

## Introduction

Deer keds (genus *Lipoptena*), also known as louse flies, are obligate, blood-feeding ectoparasites that belong to the Hippoboscidae family [1]. Deer keds typically parasitize deer, antelope, goat, and sheep [2]. After keds reach a suitable host, wings are broken off at the base, leaving behind a stump [1].

To date, ill effects by deer keds on hosts have not been well established [3]. Anemia and mechanical damage due to heavy infestation were suggested as clinical symptoms [3]. Recently, the importance of deer keds as a potential vector of various pathogens, including *Anaplasma ovis* [4], *Bartonella* spp. [5], *Rickettsia* spp. [4], and *Trypanosoma* spp. [6], was reported. Previous studies have generally investigated pathogens in *L*. *cervi*, another deer ked species, whereas pathogens in *L*. *fortisetosa* have not been well studied.

*L*. *fortisetosa* was first identified in Japan in 1965 [7], and since been identified in only a few other countries, including the Czech Republic [8], Poland [9], and Moldavia [10]. In South Korea, studies on the distribution of Hippoboscidae have identified two species of deer keds, *L*. *cervi* and *L*. *fortisetosa*, but *L*. *fortisetosa* was found only on Jeju island (33°29’N and 126°31’E), which has a warm oceanic climate [2,11,12]. However, pathogens carried by *Lipoptena* have not been well characterized.

In S. Korea, reports on vector-borne diseases and its pathogens are ubiquitous, which include anaplasmosis in human [13], *Borrelia burgdorferi* in human [14], *Bartonella* spp. in Korean water deer [15], *Coxiella burnetii* in raw milk [16], *Hepatozoon* spp. in leopard cat [17], and *Theileria* spp. in Chinese water deer [18]. Climate change, due to global warming, has engendered a more subtropical climate, which may increase the risk of vector-borne diseases nationally [19]. Warm summer seasons, in particular, provide an ideal environment for vectors throughout the country.

The objective of this study was to investigate the distribution of *L*. *fortisetosa* in inland regions of S. Korea, and to evaluate *L*. *fortisetosa* as a potential vector of pathogens including apicomplexans (*Babesia* spp., *Theileria* spp., *Hepatozoon* spp.), rickettsias (*Anaplasma* spp., *Ehrlichia* spp., *Rickettsia* spp.), *Bartonella* spp., *Borrelia* spp., and *Coxiella* spp.

## Materials and Methods

### Ethics statement

A wild Korean water deer in this study was road-killed and transferred to the Wildlife Treatment Center in Daegu. Ethical approval for the collection of keds and permission to conduct this study on this site were not required from any authority because the deer was dead when transferred to the center and removal of keds from deer was neither harmful nor against animal welfare. All the procedures regarding samplings and experiments were performed by veterinarians with appropriate handlings. The deer carcass was incinerated by an authorized company. While Korean water deer is designated as vulnerable species by International Union for Conservation of Nature and Natural Resources (http://www.iucnredlist.org/details/10329/0), the deer used in this study was dead. Thus, this study did not involve endangered or protected species.

### Collection of ked samples and species identification

Eight keds were collected from a wild Korean water deer that was road-killed at 322 Gwahakbukro (35°39'50.00" N, 128°25'34.16" E), Dalsung, Gyeongbuk province, S. Korea, in 2015 and transferred to the Wildlife Treatment Center in Daegu, S. Korea, in 2015. Species of keds were identified using morphological characteristics [1,11,20], and through analysis of the cytochrome oxidase subunit I (*cox-1*) gene using primers in Table 1.

**Table 1:** Primers used in this study to detect pathogens in deer keds (*Lipoptena fortisetosa*) collected from Korean water deer.

Species	Target gene	Name of primer	Primer sequence (5’−3’)	Expected size (bp)	Reference
*L*. *fortisetosa*	*cox-1*	CI-J-1632	TGA YCA AAT TTA YAA Y	730	Modified from [37]
		CI-N-2329	ACT GTA AAT ATR TGA TGA GCT CA		
		CI-J-1718	GGA TTT GGW AAT TGA YTA RTW CC	519	
		CI-N-2191	GGT AAA ATT AAA ATA TAA ACT TC		
*A*. *phagocytophilum*	16S rRNA	EE1	TCC TGG CTC AGA ACG AAC GCT GGC GGC	1433	[38]
		EE2	AGT CAC TGA CCC AAC CTT AAA TGG CTG		
		EE3	GTC GAA CGG ATT ATT CTT TAT AGC TTG C	928	
		EE4	CCC TTC CGT TAA GAA GGA TCT AAT CTC C		
*Borrelia* spp.	5S–23S rRNA	Bb23S3	CGA CCT TCT TCG CCT TAA AGC	412	[39]
		Bb23Sa	TAA GCT GAC TAA TAC TAA TTA CCC		
		Bb23S3nF	CTG CGA GTT CGC GGG AGA	226–266	
		Bb23SanR	TCC TAG GCA TTC ACC ATA		
*Coxiella* spp.	16S rRNA	Cox16SF1	CGT AGG AAT CTA CCT TRT AGW GG	1321–1416	[23]
		Cox16SR2	GCC TAC CCG CTT CTG GTA CAA TT		
		Cox16SR1	ACT YYC CAA CAG CTA GTT CTC A	719–813	
*Bartonella* spp.	ITS-1	QHVE-OF	TTC AGA TGA TGA TCC CAA GC	736	[15]
		QHVE-OR	AAC ATG TCT GAA TAT ATC TTC		
		QHVE-IF	CCG GAG GGC TTG TAG CTC AG	484	
		QHVE-IR	CAC AAT TTC AAT AGA AC		
*Hepatozoon* spp.	18S rRNA	HepFc	ATA CAT GWG CAM AWT CTC AAC	680	[40]
		HepRc	TTA TWA TTC CAT GCT GCA G		
*T*. *ovis*	18S rRNA	To18F	CGA ATC GCG TCT TCG GAT GCG	418	In this study
		To18R	GCC ACA ATG CAA AGA CTC G		
*T*. *luwenshuni*	18S rRNA	Tlw18F	GAA TCG CAG CTT TTG CGG CG	1061	In this study
		Tlw18R	ATA CCC GCA TCC TAT TTA GCA GG		

### DNA extraction and PCR

DNA was extracted from whole deer keds using a DNeasy^®^ Blood & Tissue Kit (Qiagen, Hilden, Germany) following the manufacturer’s instructions, and the quality and quantity of DNAs were estimated using an Infinite^®^ 200 pro NanoQuant (Tecan, Männedorf, Switzerland).

PCR assays were performed using the AccuPower PCR Premix Kit (Bioneer, Daejeon, Korea) to detect: 16S rRNA sequences of the genera, *Anaplasma* and *Coxiella*; 18S rRNA sequences of the genera, *Babesia*, *Theileria*, and *Hepatozoon*; 5S-23S rRNA region sequences of *Borrelia* spp.; and an internal transcribed spacer region sequence of *Bartonella* spp. Commercial PCR kits were also adapted to detect *Babesia* spp. (AccuPower^®^ Babesia PCR Kit, Bioneer), *Theileria* spp. (AccuPower^®^ Theileria PCR Kit, Bioneer), and rickettsias (AccuPower^®^ Rickettsiales 3-Plex PCR Kit, Bioneer) of genera, *Anaplasma*, *Ehrlichia*, and *Rickettsia*. PCR amplicons were estimated using gel electrophoresis with UV transillumination after ethidium bromide staining. The species of pathogens in deer keds were identified by designing species-specific primers, which are listed in Table 1, and comparing to expected amplicons.

### DNA sequencing

Amplicons matching expected sizes were sequenced using BigDye^®^ Terminator v3.1 Cycle Sequencing Kit (Applied Biosystems, New York, USA) following the manufacturer’s instructions, and analyzed with ABI 3730XL DNA Analyzer (Applied Biosystems). Empirical sequences were compared with those deposited in GenBank using BLASTn.

### Phylogenetic analysis

Phylogenetic relationships of *L*. *fortisetosa*, *Coxiella*-like bacteria (CLB), *T*. *luwenshuni*, and *T*. *ovis* investigated in this study were assessed using MEGA 6.06 based on a maximum likelihood method [21]. To estimate the reliability of constructed trees, bootstrap analysis was performed with 1,000 replicates. Host, country of isolation, and GenBank accession numbers are shown in the figure.

## Results

### Identification of ked species

**Fig 1:**
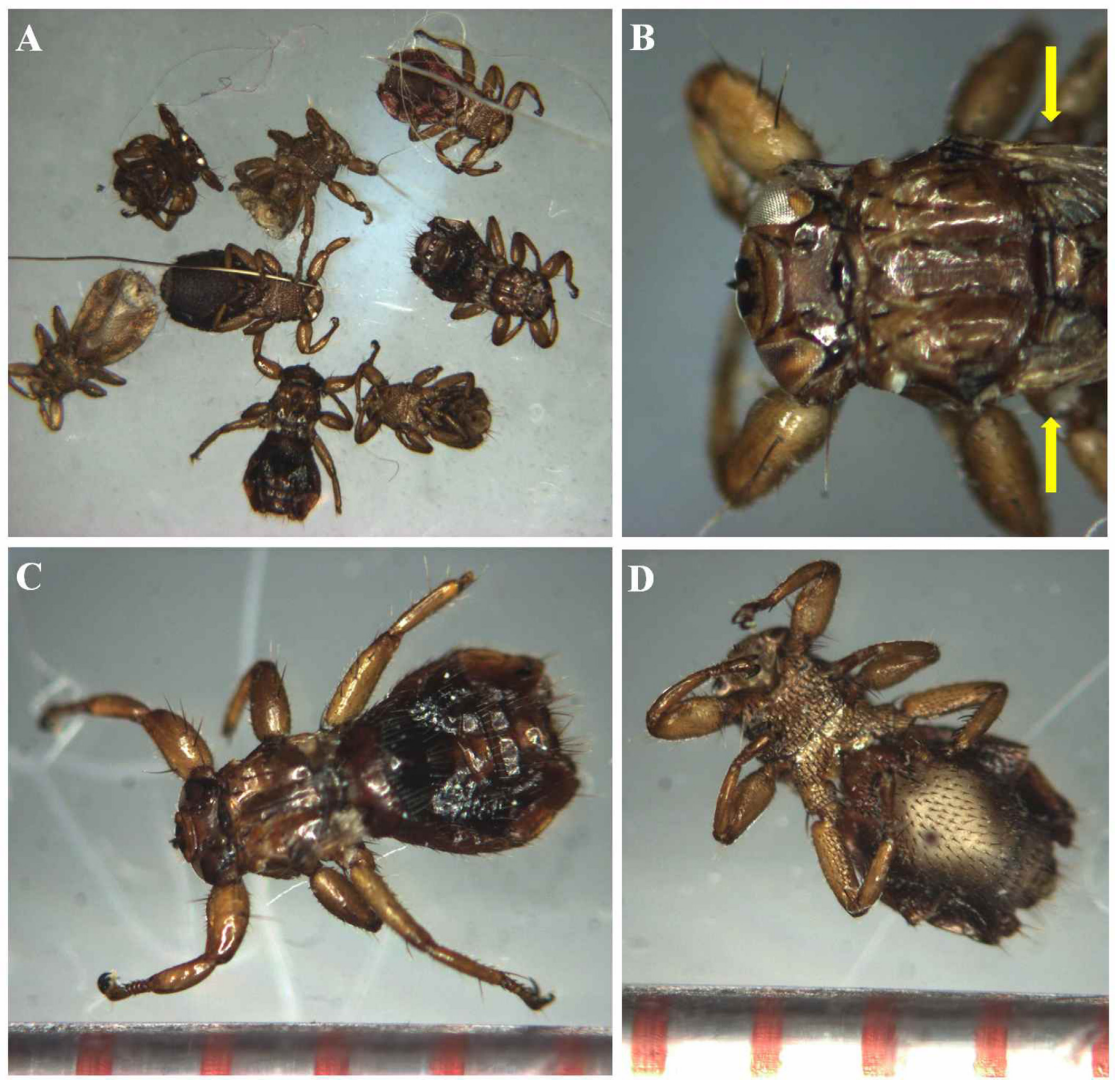
Morphology of the deer keds *Lipoptena fortisetosa* collected from a Korean water deer. (A) Eight *L*. *fortisetosa* are 3.0 mm in length. (B) Close-up of the head of *L*. *fortisetosa*: protruded proboscis at the end of the head and compound eyes. Yellow bars indicate stump of wings. (C) Dorsal view of *L*. *fortisetosa* with strong claws at the end of six segmented legs. Bodies are coved by short hair. Head and thorax parts are nearly 1.4 mm in length. (D) Ventral view of *L*. *fortisetosa*. Marks on the ruler at the bottom of the insets in C and D are 1 mm apart.

Morphologically, keds collected in this study were flattened dorso-ventrally, with a depressed head and compound eyes (Fig 1A and 1B). The thorax and abdomen gives these insects their louse-like appearance; however, wing vestiges were observed at the margin of the thorax from the dorsal view (Fig 1B, yellow arrows). In addition, the lengths of the head and thorax regions ranged between 1.3–1.5 mm, and body lengths reached ~3.0 mm (Fig 1C and 1D). These results are consistent with characteristics of *L*. *fortisetosa*, but differ from those of *L*. *cervi*. Head and thorax region lengths of *L*. *cervi* are ~2.0 mm, and body lengths are 4–7 mm [1,11,20].

**Fig 2:**
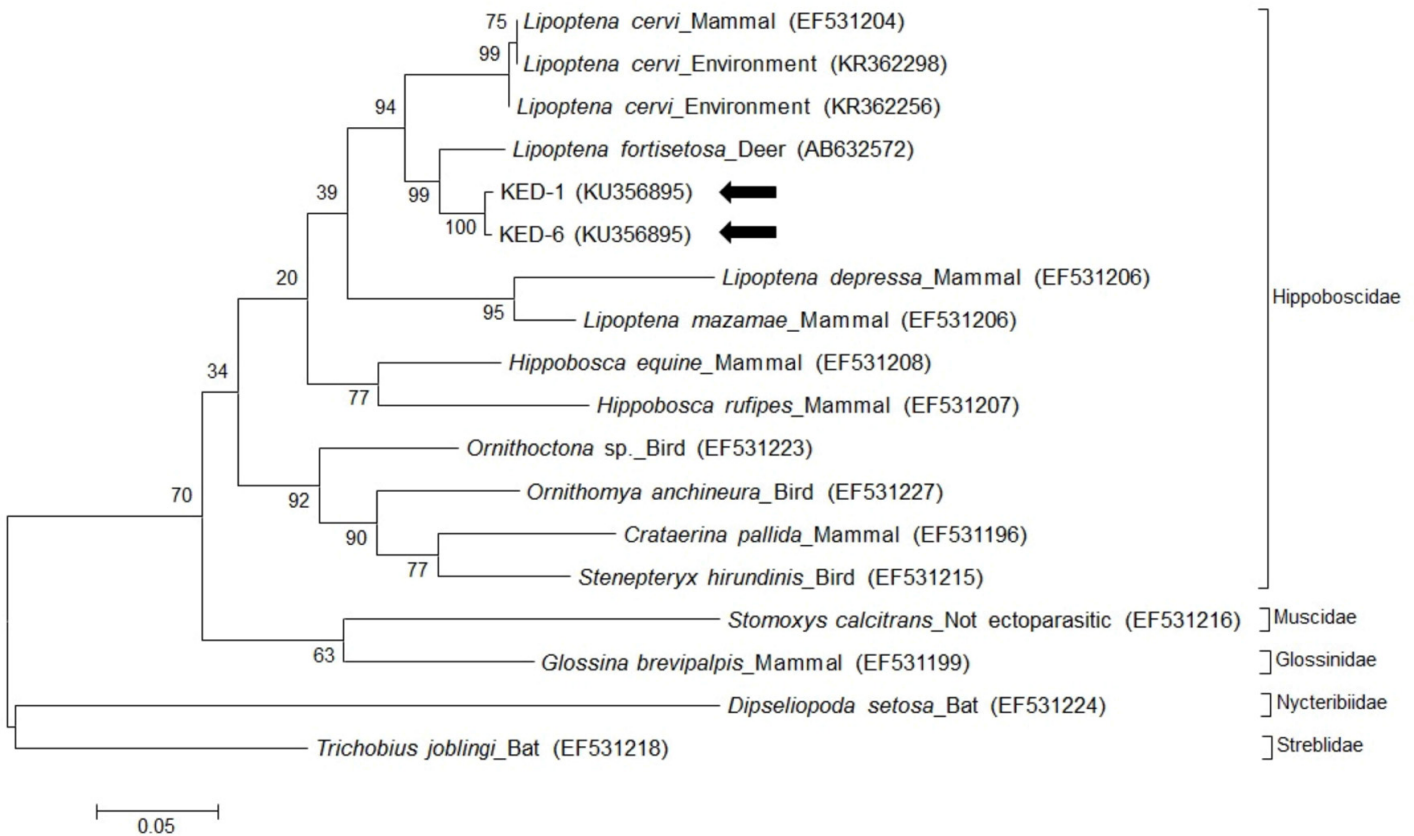
Phylogenetic analysis of the cytochrome oxidase subunit I gene in *Lipoptena fortisetosa*. The two ked sequences (KED-1, 6) are marked by arrows. Phylogenetic trees were constructed based on the maximum likelihood method with 1,000 replicates. Scale bar represents the phylogenetic distance between sequences. Species, host, and GenBank accession numbers are included in the figure.

Moreover, universal primers for the *cox-1* gene were used to amplify 472-bp fragments from ked samples. The sequences (accession nos. KU356895 and KU356896) of the *cox-1* gene in two ked samples exhibited 95.4% and 96.0% identity, respectively, with the *cox-1* gene of *L*. *fortisetosa* (accession no. AB632572) deposited in GenBank (Fig 2). Based on morphological characteristics and *cox-1* sequence identity, keds collected from a Korean water deer were identified as *L*. *fortisetosa*.

### PCR and phylogenetic analysis

In all, three species of endosymbionts in *L*. *fortisetosa* were identified by PCR. *Coxiella* spp. were detected in five keds, and *T*. *luwenshuni* and *T*. *ovis* were detected in six keds. None of the rickettsias, *Babesia* spp., *Bartonella* spp., *Borrelia* spp., and *Hepatozoon* spp. was detected (Table 2). Individual keds carried from zero to three endosymbionts.

**Table 2:** Pathogens detected in *Lipoptena fortisetosa* using PCR.

ID of ked	KED-1	KED-2	KED-3	KED-4	KED-5	KED-6	KED-7	KED-8	Total
*Coxiella* spp.	+	+	+	+	+	-	-	-	5/8 (62.5%)
*Theileria ovis*	+	+	+	+	-	-	+	+	6/8 (75.0%)
*Theileria luwenshuni*	+	+	+	+	-	-	+	+	6/8 (75.0%)
No. of pathogens detected	3	3	3	3	1	0	2	2	

**Fig 3:**
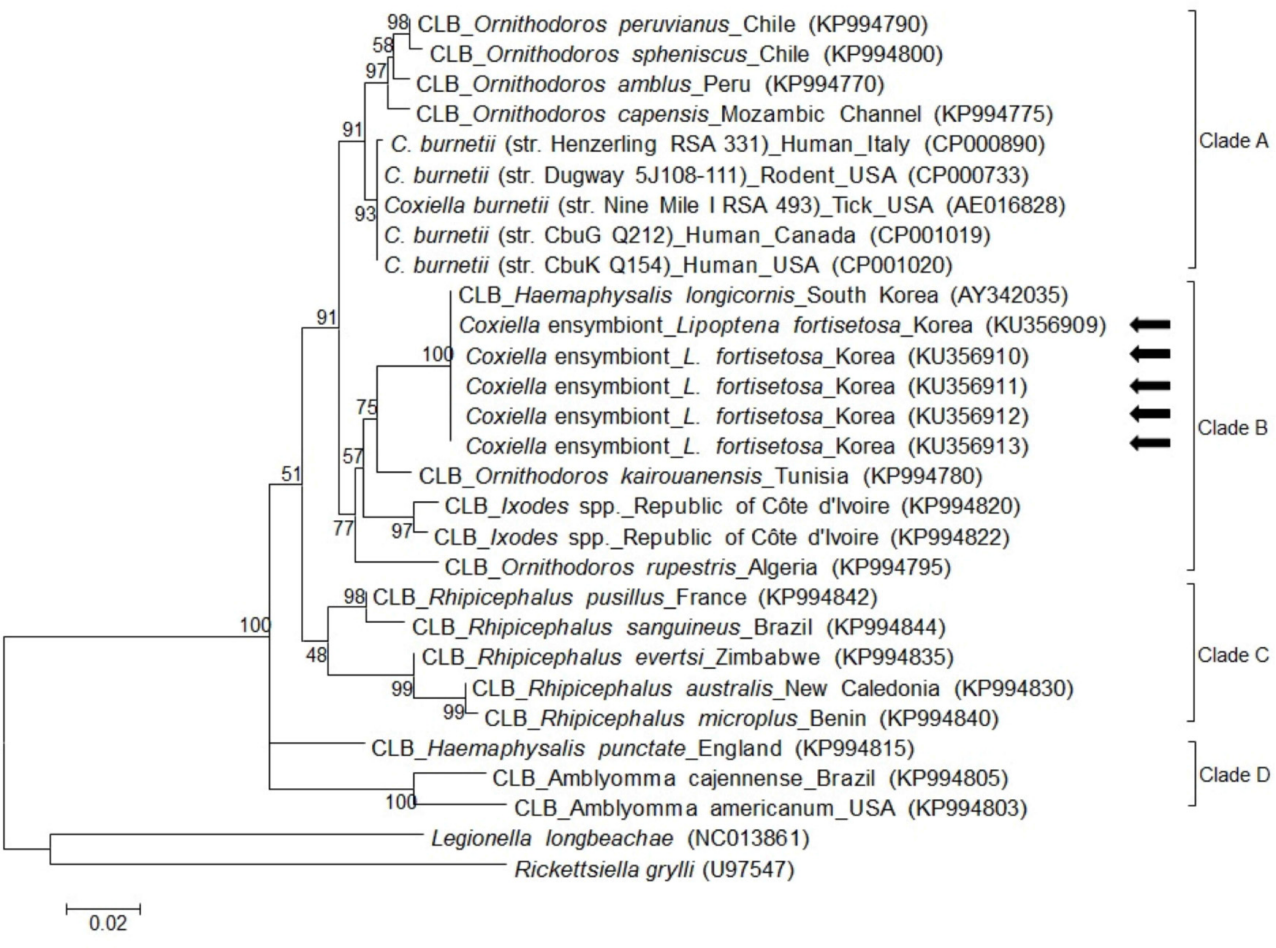
Phylogenetic analysis of *Coxiella* 16S rRNA in *Lipoptena fortisetosa*. All 5 *Coxiella*-like bacteria (CLB) in this study showed 100% identity with one another and with the *Coxiella* endosymbiont (AY342035) of *Haemaphysalis longicornis* in South Korea. CLB in this study are marked with arrows. Phylogenetic trees were constructed based on the maximum likelihood method with 1,000 replicates. Scale bar represents the phylogenetic distance between sequences. Species, host, region of isolation, and GenBank accession numbers are included in figure.

For *Coxiella* spp., 719-bp fragments of 16S rRNA were amplified. All five of the obtained sequences exhibited 100% identity to each other, and 99.4% identity with a *Coxiella* endosymbiont sequence from *Haemaphysalis longicornis* in S. Korea (AY342035) deposited in the GenBank database. Based on a previous study [22], CLB detected in this study were classified into clade B (Fig 3).

**Fig 4:**
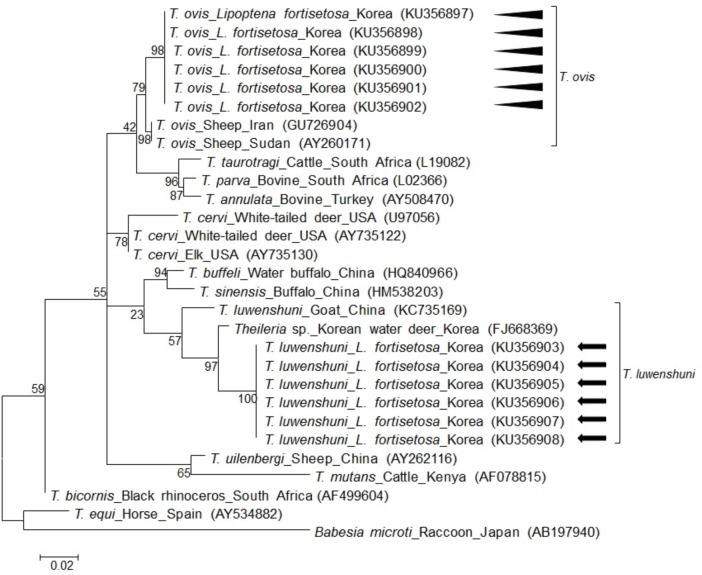
Phylogenetic analysis of the 18S rRNA in *Theileria luwenshuni* and *T*. *ovis* in *Lipoptena fortisetosa*. All 6 *T*. *luwenshuni* detected in this study showed 100% identity with one another and with the *Theileria* sp. (FJ668369) identified from Korean water deer. All 6 *T*. *ovis* detected in this study showed 100% identity with one another and with *T*. *ovis* (GU726904) identified from a sheep in Iran. The sequences of *T*. *luwenshuni* and *T*. *ovis* are marked with an arrow and arrowhead, respectively. Phylogenetic trees were constructed based on the maximum likelihood method with 1,000 replicates. Scale bar represents the phylogenetic distance between sequences. Species, host, region of isolation, and GenBank accession numbers are included in the figure.

Using *Theileria* genus-specific primers, 259-bp fragments of *Theileria* 18S rRNA were amplified. However, the sequences of 259-bp amplicons were insufficient to differentiate among species since sequences were highly similar among *T*. *cervi* (100%, GU946217), *T*. *luwenshuni* (99.6%, KC769997), and *T*. *ovis* (100%, JX262363). After designing species-specific primers for *T*. *cervi*, *T*. *luwenshuni*, and *T*. *ovis*, 420-bp and 1058-bp gene fragments for 18S rRNA of *T*. *ovis* and *T*. *luwenshuni* were amplified, respectively, whereas no amplicons were detected for *T*. *cervi*. Both *T*. *ovis* and *T*. *luwenshuni* were detected in six keds. For each species, the sequences were 100% identical (Fig 4). When compared with sequences in GenBank, *T*. *ovis* shared 98.8% identity with *T*. *ovis* (KP019206), and *T*. *luwenshuni* shared 98.7% identity with *T*. *luwenshuni* (KC735157).

The obtained sequences in this study were submitted to GenBank. Accession numbers are KU356897–KU356902 (*T*. *ovis*), KU356903–KU356908 (*T*. *luwenshuni*), and KU356909–KU356913 (CLB).

## Discussion

*Coxiella* genus includes *C*. *burnetii*, *C*. *cheraxi*, and unclassified CLB [22,23]. Among them, *C*. *burnetii* is a zoonotic pathogen that causes acute or chronic illness and flu-like symptoms in humans, and abortion in animals [23]. *C*. *burnetii* is shed in milk, feces, and urine from infected animals and can be transmitted by inhalation of aerosolized microorganisms [24]. Due to its environmental resistance, route of transmission, and difficulty to diagnose, *C*. *burnetii* is designated as a category B potential biological weapon by the United States [25].

According to a recent study, *Coxiella* spp. could be classified into four different clades (A–D), according to their genetic characteristics [22], with *C*. *burnetii* belonging to clade A. In this study, CLB detected from *L*. *fortisetosa* was classified into clade B through genetic analysis of 16S rRNA. In addition, CLB detected in our study exhibited 100% identity with *Coxiella* endosymbiont (AY342035) from *H*. *longicornis* in S. Korea. The perfect sequence identity of 16S rRNA between CLB in this study and AY342035 in a previous study suggests that CLB is potentially transmitted between mammals by ticks and keds.

In previous studies, *C*. *burnetii* and CLB were detected from various sources including ticks, flies, dairy cattle, raw milk, and aborted fetuses [23,24,26]. In 1958, *C*. *burnetii* was detected from *Melophagus ovinus* (Family: Hippoboscidae), also known as sheep ked, and confirmed as a bona fide vector of *C*. *burnetii* through animal experiments, clinical manifestations, and antigen-antibody tests [27]. Based on these results, we suggest that *L*. *fortisetosa* could act as a vector not only for CLB but also for *C*. *burnetii*, though further studies are required to confirm this hypothesis. Although the virulence of CLB to vertebrates is still controversial [23], several studies have reported clinical symptoms (systematic and even fatal) in birds due to CLB infections [28–30]. Therefore, further studies are required to assess the virulence of CLB to vertebrate.

Till now, different *Theileria* spp. including *T*. *lestoquardi*, *T*. *ovis*, *T*. *uilenbergi*, *T*. *luwenshuni*, *T*. *separate*, and *T*. *recondite* have been found in small ruminants [31]. Of these species, *T*. *lestoquardi*, *T*. *luwenshuni*, and *T*. *uilenbergi* are known to be highly pathogenic to sheep and goats [31]. In S. Korea, *T*. *luwenshuni* and *T*. *ovis* were reported in Chinese water deer [18], and *T*. *luwenshuni* was found in roe deer and *H*. *longicornis* [32]. In the past, *T*. *luwenshuni* was considered to be a *Theileria* sp. indistinct from *T*. *ovis* and *T*. *lestoquardi* that caused ovine and caprine theileriosis [33]; however, it can now be distinguished from *T*. *ovis* and *T*. *lestoquardi* based on their biological characteristics and 18S rRNA sequence [33]. This is particularly important as the pathogenicities of *T*. *ovis* and *T*. *luwenshuni* to ruminants vary. In this study, 259-bp of *Theileria* 18S rRNA was amplified by PCR but amplicon was insufficient to clearly differentiate the species, owing to the high sequence similarity among *Theileria* spp. Using species-specific primer sets, we revealed the presence of mixed infection of *T*. *ovis* and *T*. *luwenshuni* in *L*. *fortisetosa*.

The sequences of *T*. *luwenshuni* in this study showed 100% identity to a *Theileria* sp. (FJ668369) that was detected from a Chinese water deer in S. Korea [18], and 98.7% identity to *T*. *luwenshuni* (KC735157) from a goat in China. While the sequence FJ668369 was originally submitted simply as *Theileria* sp., we now propose that species is *T*. *luwenshuni* based on our phylogenetic analysis. The sequences of *T*. *ovis* in this study showed 98.8% identity to *T*. *ovis* (FJ668373) that was detected from a Chinese water deer in S. Korea [18]. The high sequence similarity between these sequences and sequences obtained in previous studies suggest the potential transmission of *Theileria* among mammals, ticks, and keds.

Deer, antelope, goats, and sheep are the main hosts of deer keds [2]. However, incidental infestation of deer keds in other animals, including dogs, horses, and other ruminants, has also been reported [34]. Moreover, human dermatitis caused by ked bites has been often reported in Finland [35].

Recently, due to global warming and an increasing number of wild animals in S. Korea, caution to vector-borne diseases has been raised. Vectors generally transmit pathogens mechanically or biologically [36]. The results of this study suggest that *L*. *fortisetosa* is a potential biological vector of CLB endosymbionts. However, ecological aspects, such as the life cycle and reproduction of deer keds, are yet to be determined.

In this study, we report the first identification of CLB, *T*. *ovis* and *T*. *luwenshuni* in *L*. *fortisetosa*. Further investigations are required to confirm *L*. *fortisetosa* as a biological vector of these pathogens. Moreover, owing to the possible transmission of vector-borne pathogens, members of the medical and veterinary field need to be cautious of potential contact with deer keds.
